# Whole-Body Cryotherapy Is an Effective Method of Reducing Abdominal Obesity in Menopausal Women with Metabolic Syndrome

**DOI:** 10.3390/jcm9092797

**Published:** 2020-08-30

**Authors:** Magdalena Wiecek, Jadwiga Szymura, Justyna Sproull, Zbigniew Szygula

**Affiliations:** 1 Department of Physiology and Biochemistry, Faculty of Physical Education and Sport, University of Physical Education in Krakow, 31-571 Kraków, Poland; 2 Department of Clinical Rehabilitation, Faculty of Motor Rehabilitation, University of Physical Education in Krakow, 31-571 Kraków, Poland; jadwiga.szymura@awf.krakow.pl; 3 Ph.D. Studies, Faculty of Physical Education and Sport, University of Physical Education in Krakow, 31-571 Kraków, Poland; bednarekjustyna1@gmail.com; 4 Institute of Biomedical Sciences, Faculty of Physical Education and Sport, University of Physical Education in Krakow, 31-571 Kraków, Poland; wfszygul@cyf-kr.edu.pl

**Keywords:** whole-body cryotherapy, body composition, visceral obesity, menopausal women, metabolic syndrome, irisin, interleukin-6

## Abstract

Abdominal obesity predominates in menopausal women (MW) and contributes to the development of metabolic syndrome (MetS). It is associated with increased mortality related to cardiovascular disease, diabetes and fatty liver disease. The effects of whole-body cryotherapy (WBC) on body composition and the blood concentration of irisin, interleukin-6 (IL-6) and C-reactive proteins (CRP) in MW with MetS and in healthy women (HW), were assessed. The study included 19 women with MetS (61.53 ± 3.99 y, BMI 30.09 ± 4.98 kg/m^2^) and 18 HW (60.28 ± 3.63 y, BMI 25.50 ± 2.37 kg/m^2^) who were subjected to 20 WBC treatments at −130 °C for 3 min daily. In both groups, body mass (BM), BMI, abdominal circumference, triceps skinfold, total fat mass and percentage of leg fat significantly decreased after 20 WBC sessions. Additionally, the percentage of total, trunk and android fat in the MetS group were significantly decreased after 20 WBC applications. Waist circumference (WC) and waist-to-height ratio (WHtR) significantly decreased in both groups, and in the HW group, hip circumference and abdominal skinfold also significantly decreased after 10 WBC and 20 WBC treatments. In both groups, the concentration of plasma irisin significantly increased after 1 WBC and 10 WBC exposures, while the concentration of IL-6 significantly increased only in MetS group after 10 WBC and 20 WBC, and were significantly higher than in HW. CRP concentrations were significantly higher in the MetS group than in HW before 1 WBC, after 1 WBC and 10 WBC sessions, but not after 20. In the MetS group, there were significant negative correlations between the change in irisin level and the changes in WC and BM, and between the level of irisin and the change in percentage of total fat, and significant negative correlations between the change in IL-6 level and changes in WC, waist-to-hip ratio and WHtR. Whole-body cryotherapy, assuming the application of 20 treatments in the series, reduces abdominal obesity in menopausal women indirectly through the secretion of irisin and IL-6, and can be used as adjunctive therapy in the treatment of metabolic syndrome. Our conclusion is limited to menopausal women with low–moderate physical activity for whom its level as well as diet were not changed during the treatment.

## 1. Introduction

Obesity, defined as abnormal or excessive fat accumulation that presents a risk to health, is a serious global problem [1]. Overweightness and obesity are more common in women [2,3]. Globally, the prevalence of overweightness (BMI ≥ 25 kg/m^2^) and obesity (BMI ≥ 30 kg/m^2^ in adults has increased among men from 25.4% in 1980 to 38.5% and 10.1% (respectively) in 2015, while among women, from 27.8% and 8.9% to 39.4% and 14.8%, respectively [3]. It has been estimated that by 2025, the percentage of obese males world-wide will total 18%, and for women, 21% [2]. The highest percentage of obese women occurs in the menopausal period [3]. Menopausal women are most often characterised by abdominal obesity, also known as android or visceral obesity [4,5]. In menopausal women, the increase in BMI and total body mass, which consists of fat mass and the remaining tissues (lean body mass), is mainly due to an increase of fat in the abdominal area. In this period, the amount of visceral fat among women increases by over 40%, and the amount of subcutaneous fat in the abdominal region by about 20% [6]. In population studies carried out in the USA and Poland, it has been shown that over 60% of menopausal women were characterised by abdominal obesity [7,8]. Abdominal obesity is a major cause of cardiovascular disease, diabetes, musculoskeletal disorders, especially osteoarthritis, and some cancers (including endometrial, breast, ovarian, prostate, liver, gallbladder, kidney, and colon) [1].

Abdominal obesity contributes to the development of metabolic syndrome (MetS) [9], in which dyslipidemia, hyperglycaemia and arterial hypertension are also diagnosed [10,11,12,13]. It is estimated that the occurrence of MetS in Europe, although varying in different countries, affects about 37.6% of women aged 60–78, while in men, this value is 21.8% [14,15]. In Poland, MetS is present in approximately 46% of women and 34% of men aged 60–74 [16]. The most common diagnostic criterion was abdominal obesity, which was more common in women than in men, in 40.0% and 29.1% of people, respectively [17].

Abdominal obesity leads to an imbalance in the production and secretion of pro-inflammatory (IL-6, IL-1β, TNF-α) and anti-inflammatory (IL-10) factors, and contributes to the occurrence of chronic, low-grade systemic inflammation [18,19]. The concentration of acute phase inflammatory protein like C-reactive protein (CRP) and markers of oxidative damage is higher in obese people and positively correlates with BMI, percentage of adipose tissue and triglyceride levels [20].

The epidemic nature of obesity and its consequences is a major worldwide, public health problem [14,15,16,17,21,22,23]. There are studies on the effectiveness of using whole-body cryotherapy as a non-pharmacological method of supporting the treatment of obesity and metabolic disorders [24,25,26,27,28].

Whole-body cryotherapy (WBC) consists in short-term (1–3 min), repeated exposure to low temperatures (from −110 °C to −160 °C) on the largest possible surface of exposed skin [29]. It has been shown that WBC treatments cause a generalised physiological response of the body [30]. According to the International Classification of Medical Procedures (ICD-9), WBC is a physiotherapeutic treatment (93.3950). In Poland, WBC treatments belong to medical services used as treatments preceding kinesiotherapy with analgesic, anti-swelling and anti-inflammatory effects, mainly in rheumatoid and neurological diseases. As treatments reimbursed by the National Health Fund (13/2019/DSOZ) [31], they can be applied 6 months apart, each in the number of 10 treatments, daily on working days in 2 consecutive series. In people qualified for the procedures, there are no contraindications for using more WBCs in a series and their more frequent repetition. In scientific research, WBC is most often used once a day, applying a total of 5 to 30 treatments [29].

Depending on the number of treatments, duration, frequency and temperature range, various metabolic effects of these treatments are possible [30]. There is a need to study the distant effects of WBC application. To our knowledge, only Szymura et al. [32] showed an increase in IL-3 and erythropoietin levels 7 days after the completion of a series of 24 WBC treatments in older marathon runners and non-trained men. Although not all research results are consistent, it has been demonstrated that WBC may have a beneficial effect on hormonal and metabolic changes, and is also anti-inflammatory [29,30]. An increase, inter alia, has been noted in the concentration of IL-6, without changes in the concentration of TNF-α in the blood after WBC procedures [33], which indicates an anti-inflammatory effect and may, via IL-6, positively influence metabolism [34,35]. Improvement in lipid profile [24,25] and a decrease in the concentration of resistin, visfatin [24] and asprosin [27] were found in the blood of obese individuals as a result of WBC application. However, there was no effect of WBC on changes in leptin and adiponectin levels [24,27]. Obese men characterised by low physical activity showed an increase in irisin concentration as a result of exposure to cryogenic temperatures [26].

Irisin is a 29-amino acid fragment of the extracellular domain of the FNDC5 protein, the gene of which is expressed mainly in myocytes [36] and adipocytes [37]. A negative correlation was found between the concentration of irisin in the blood and age [38]. Irisin affects the browning of white adipose tissue; by inducing UCP-1 expression, it increases energy expenditure as a result of non-shivering thermogenesis; influences glucose homeostasis and improves insulin sensitivity [39]. In an animal model, a protective role of irisin against obesity has been demonstrated by reducing the concentrations of LDL, TG, glucose and leptin in the blood [40]. People with MetS demonstrated lower levels of irisin and, at the same time, higher levels of CRP and IL-6 in the blood compared to individuals without MetS. Lower levels of irisin were found in people with high CRP, but not in those with high IL-6 [41].

It is known that physical exercise is a factor increasing irisin secretion in obese people [42]. In two earlier conducted studies, it was indicated that exposure to cryogenic temperatures may also be a factor inducing the secretion of this hormone, although the studies were carried out only in men, both with normal body mass [43] and those obese [26].

The aim of our study is to evaluate the effect of 10 and 20 WBC sessions performed daily in series of 5 on irisin secretion and body composition in menopausal women with and without metabolic syndrome.

Taking into account the metabolic effect of irisin and the induction of the effect of cryogenic temperatures on the secretion of this hormone in obese men, as shown in previous studies [26], and the induction of IL-6 secretion, without intensifying inflammation [33], we hypothesise that 20 WBC treatments reduce the content of adipose tissue and abdominal obesity in menopausal women, which is indirectly associated with an increase in the concentration of irisin and IL-6 in the blood.

## 2. Methods

### 2.1. Study Design

As in the previous study [27], Caucasian menopausal women (no menstruation for at least 12 months) aged 55–70 years, who had no contraindications for the application of WBC, were included in the study [29]. People who had participated in WBC procedures within the last 6 months, as well as smokers, alcohol abusers, chronically ill patients, those taking medications on a permanent basis, except for antihypertensive therapy, people whose diet deviated from the standards developed for the Polish population by the Institute of Food and Nutrition [44], e.g., following specific diets (vegetarian, vegan, diabetic, low-calorie) or dietary supplements that may affect the metabolic rate, were also excluded from the trial.

The first stage of the study included internal medicine examination, during which the presence of metabolic syndrome was diagnosed, taking into account the criteria proposed by the National Cholesterol Education Program-Adult Treatment Panel III (NCEP-ATP III) [10,11,12,13] as well as cardiological and gynaecological examinations.

MetS was diagnosed in people who met at least 3 of any of the following criteria:

- Waist circumference > 88 cm;
- TG concentration ≥ 150 mg/dL;
- HDL concentration < 50 mg/dL;
- fasting glucose ≥ 100 mg/dL; and
- systolic blood pressure (SBP) ≥ 130 mmHg o diastolic blood pressure (DBP) ≥ 85 mmHg or anti hypersensitive therapy.

During the second stage, a series of 20 WBC treatments was performed. Somatic measurements (body mass, waist, abdominal and hip circumferences, skin fold thickness), body composition assessment and biochemical determinations (irisin, IL-6, CRP) were performed in fasting state before the 1st WBC, the day after the 10th WBC and after the 20th WBC treatments. Biochemical determinations were also performed the day after WBC 1.

Physical activity and diet were evaluated. The subjects were asked to maintain their current physical activity and diet, and not to undergo any biological regeneration treatments (i.e., massage, sauna, hydrotherapy) while using WBC.

The study was conducted in accordance with the Declaration of Helsinki. The methodology of the study was approved by the Bioethical Committee of the Regional Medical Chamber (96/KBL/OIL/2015, 3 July 2015).

### 2.2. Participants

The inclusion criteria were met by 41 out of the 79 volunteers, and 20 were diagnosed with MetS. During the period of using WBC treatments, 4 people resigned from the study, including 2 for organisational and 2 for health reasons (upper respiratory tract infection and extensive cut of the facial skin as a result of a fall not related to the use of WBC). The entire study programme was completed by 37 volunteers, 19 of whom had MetS, the remaining women were healthy and controlled. In the study group, 7 women exhibited normal body mass (BMI 23.4 ± 2.29 kg/m^2^), 22 were overweight (27.48 ± 2.51 kg/m^2^) while 8 were obese (BMI 32.76 ± 6.05 kg/m^2^). The characteristics of the participants, according to the MetS and healthy division, are presented in Table 1.

The most frequently repeating disorders among the criteria adopted in the diagnosis of metabolic syndrome among the study group were hyperglycaemia, abdominal obesity and hypertension. Detailed results according to group are presented in Table 2.

### 2.3. Whole-Body Cryotherapy

The examined women underwent 20 WBC sessions, which were performed daily in 4 series of 5 procedures, with a 2-day interval between the series [27]. Treatments were performed at a professional medical cryotherapy centre in a liquid nitrogen-cooled cryogenic chamber, consisting of a vestibule (−60 °C) connected directly to the main chamber (−130 °C).

The chamber was equipped with a system for automatic temperature monitoring and air dehumidification, as well as with oxygen sensors. During the procedure, continuous contact with the subjects was ensured through the audiovisual system, as well as eye contact through thermal glass in the chamber door. In order to ensure safety during the procedure, both in the proper chamber and in the vestibule, there were alarm buttons and mechanical levers enabling the user to immediately open the door from inside. Treatments were performed under the supervision of qualified physical therapists.

Before beginning the WBC series, the volunteers were informed in detail about how to prepare for the procedure and how to behave during its implementation.

During the procedures, the women wore a sleeveless cotton tops and shorts that did not put pressure on the skin; the clothes were devoid of rigid, including metal, elements. Additionally, they were equipped with a surgical mask with an additional layer of gauze covering the nose and mouth, woollen socks protecting the ankle joints and knee pads, gloves, a band or a cap protecting the auricles, and clogs with a wooden sole. The women did not apply cosmetic products to the skin before entering the cryochamber and thoroughly dried the skin to remove any sweat, the presence of which could cause frostbite. Before initiating the WBC procedure, the women took off any jewellery or glasses, and did not use contact lenses.

Each procedure consisted of a 30-second stay in the vestibule of the chamber, followed by a 3 min stay in the main chamber. Up to 4 people participated in the procedure at a time. During their stay in the main chamber, the women walked around slowly, changing their direction every 30-second at the signal. The women were asked to breathe calmly during the procedure, inhaling through their noses, and exhaling through their mouths, and not to adjust their clothes or touch exposed parts of the body.

Blood pressure was measured before each treatment. In none of the cases did the measurements exceed 150/90 mmHg, which was adopted as the borderline value allowing for carrying out the WBC procedure [45]. In addition, blood pressure measurements were also taken after 1, 10 and 20 WBC treatments. There was no significant effect of single application or a series of 10 and 20 treatments on systolic or diastolic blood pressure.

### 2.4. Somaticmeasurements and Body Compositionevaluation

Body height was measured to the nearest 1 mm using a stadiometer (Seca 217, Hamburg, Germany).

Body mass (BM) was measured while standing in underwear (Jawon IOI-353 Body composition Analyzer, Gyeongsa, Korea).

Circumference was measured in a standing position to the nearest 1 mm, placing an anthropometric tape (Seca 201, Hamburg, Germany) perpendicular to the vertical axis of the body. Waist circumference (WC) was measured at the narrowest point between the lower edge of the costal arches and the highest point of the iliac crests. Abdominal circumference (AC) was measured approximately 1 cm below the navel. Hip circumference (HC) was measured above the buttocks at the widest point around the greater trochanter, making sure not to be lower than the pubic symphysis. Waist and abdominal circumferences were measured at the end of calm exhalation.

Skin fold thickness was measured in a standing position using the calliper method to the nearest 0.1 mm (Harpenden skinfold calliper, Burgess Hill, UK) on the abdomen, at the lower angle of the scapula and above the triceps muscle of the upper limb.

All measurements of circumferences and skin fold thickness were taken 3 times by the same person with extensive experience, always using the same anthropometric tape and the same calliper. In the analysis, the average of the two closest results was taken into account.

Body composition was measured by dual energy X-ray absorptiometry (DXA) (GE Healthcare Lunar iDXA, Madison, WI, USA) which, according to the manufacturer’s instructions, was calibrated each day before the series of measurements. During the measurement, the women wore clothes that did not restrict the waist area (cotton sleeveless top and shorts), and were asked to remove plastic, rubber, metal objects and jewellery. The examination was performed in supine, immobile position, with the upper limbs extended along the body, the palms of the hands pointing towards the thighs. During the examination, segmental assessment of body composition was performed.

For each of the participants, Body Mass Index (BMI), Waist-Hip Ratio (WHR) and Waist-Height Ratio were calculated according to the formulae:

BMI = body mass (kg)/(body height (m))^2^ (1)

WHR = waist circumference (cm)/hip circumference (cm)(2)

WHtR = waist circumference (cm)/body height (cm)(3)

### 2.5. Biochemical Analysis

The concentrations of IL-6 (EDTA) and irisin (EDTA and aprotinin 0.6 TIU/1 mL of blood) were determined in the plasma, and the concentration of CRP in the serum (clotting activator). Venous blood was collected using a vacuum system (Becton Dickinson, Franklin Lakes, NJ, USA) and centrifuged (RCF 1.000× *g*) for 15 min at 4 °C (MPW-351R, MPW Med. Instruments, Warsaw, Poland). The supernatant was stored until analysis at −70 ± 5 °C (ZLN-UT 300 PREM, POL-EKO-APARATURA, Wodzislaw Slaski, Poland).

The concentrations of IL-6 and irisin were determined via enzymatic immunoassay (ELISA), according to manufacture guidelines, using the highly sensitive HS600B test (R&D Systems, Inc., Minneapolis, MN, USA) and Irisin/FNDC5 EK-067-16 (Phoenix Pharmaceuticals, Inc., Burlingame, CA, USA). The results were read from a standard curve created during each of the determinations. The sensitivity of the assay for IL-6 was 0.039 pg/mL, while the intra-assay CV < 7.8%, and the inter-assay CV IL-6 < 7.2%. The detection range for irisin was 0–100 ng/mL. CRP concentration was determined via the immune turbidimetric method using the Cardiac C-Reactive Protein (Latex) High Sensitive Test-CRPHS (Roche Diagnostics GmbH, Mannheim, Germany). The detection range of the CRPHS assay was 0.15–20 mg/L, while for the inter-assay, CV < 10%.

### 2.6. Assessment of Physicalactivity

Before the first application and in the last week of using WBC, the women’s physical activity (PA) was assessed using the short version of the International Physical Activity Questionnaire (IPAQ)-Polish version, according to the scale: high, moderate or low. The IPAQ contains 7 questions on PA related to everyday life, work and leisure, performed within the 7 days preceding the survey [46].

Prior to WBC, it was found that 11 people (58%) in the MetS group exhibited moderate PA, while 8 (42%) demonstrated low PA. In the group of healthy women, 9 participants were characterised by moderate (50%) physical activity, and for 9, this level was low (50%). The PA did not change significantly during the period WBC implementation. At the end of the study, moderate PA was declared by 10 people in the MetS group and 8 healthy people, while low PA was affirmed by 12 and 10 people in the MetS and healthy groups, respectively. None of the women demonstrated high PA.

### 2.7. Assessment of Nutritional Behaviour

Since the change in caloric content and composition of the diet can significantly affect the change in body composition [47] and in the level of irisin [48,49], the volunteers were asked not to change their diet and to record the composition of the meals consumed on a daily basis. The portion size was determined based on a photo album of products and dishes [50]. Nutrient and energy consumption were calculated (Dieta 5.0, Institute of Food and Nutrition, Warsaw, Poland). The menus were assessed in relation to the nutritional standards for the Polish population developed by the Institute of Food and Nutrition [44]. The daily (24-h) dietary caloric intake before the 1st session WBC was 1911.85 ± 385.48 kcal/d and 1735.26 ± 333.72 kcal/d, respectively in the MetS group and in healthy women. The daily supply of protein, carbohydrates and fat in the diet before WBC1 in the MetS group was: 16.22 ± 4.14%, 54.31 ± 12.28% and 27.19 ± 9.21%. In the group comprising health women, these values totalled: 17.20 ± 4.35%, 54.58 ± 11.17% and 25.68 ± 7.32%, respectively. The menus from the week before beginning WBC treatments (Pre 1 WBC) and the week before the 10th (Pre 10 WBC) and 20th WBC (Pre 20 WBC) procedures were assessed. There were no differences in the caloric or main nutrient intake within or between groups during the study period (*p* > 0.05).

### 2.8. Statistical Analysis

The distribution of results for the analysed variables was checked using the Shapiro–Wilk test, and the equality of variance with the Levene test. For single measurements, the significance of group-related differences was assessed using independent-sample tests, Student’s *t*-test or the Mann–Whitney U test. Pearson or Spearman correlations were calculated.

Comparing the impact of WBC treatments on changes in the analysed variables among the compared groups, analysis of variance with repeated measures (ANOVA) was used, examining the influence of the main factors, i.e., Group (MetS and Healthy), Treatment (influence of WBC) and Group × Treatment interaction. Effect sizes for ANOVA analysis were calculated using partial eta squared (η^2^) and interpreted as 0.010–0.059 = small, 0.060–0.139 = medium, ≥0.14 = large. For changes in the level of specific variables after WBC, confidence intervals were determined (95% CI). When significant influence of the main factors was found, post-hoc analysis was performed using the Fisher LSD test. The statistical significance of differences was assumed for the level of *p* < 0.05. The statistical power of the test was also calculated (1-β). The STATISTICA 13 package (StatSoft, Inc., Tulsa, OK, USA) was used for calculations.

## 3. Results

### 3.1. Changes in Body Composition, Circumferences and Skin Fold Thicknesses as an Effect of Applying Whole-Body Cryotherapy Procedures

#### 3.1.1. Body Mass and Composition

A significant effect of WBC was noted (ANOVA Treatment, large effect size) on changes in BM (η^2^ = 0.17, *p* = 0.001), BMI (η^2^ = 0.17, *p* = 0.001) and total body fat mass (η^2^ = 0.19, *p* = 0.001), as well as in the percentage value of fat in the lower limbs (η^2^ = 0.14, *p* = 0.006), while a medium effect size was observed for changes in percentage value of fat content in total body mass (η^2^ = 0.09, *p* = 0.036) as well as in the abdominal (android) area (η^2^ = 0.08, *p* = 0.049) and trunk (η^2^ = 0.06, *p* = 0.113) (Table 3).

After 20 WBC treatments, in the MetS group and in the group of healthy women, significant reductions were noted (post-hoc, *p* < 0.05) in BM (−0.56: 95% CI −1.20; 0.08 and −0.67: 95% CI −0.98; −0.35, respectively), BMI (−0.24: 95% CI −0.49; 0.02 and −0.25: 95% CI −0.37; 0.14, respectively) and total fat mass (−0.50: 95% CI −0.93; −0.07 and −0.51: 95% CI −0.89; −0.12, respectively), as well as in the percentage of fat in the lower limbs (−0.55: 95% CI −1.01; −0.09 and −0.51: 95% CI −1.10; 0.09, respectively). Additionally, in the MetS group, after 20 WBC sessions, the percentage of adipose tissue in the total body mass decreased (−0.48: 95% CI −0.91; −0.05) significantly (post-hoc, *p* < 0.05) as well as in the trunk (−0.64: 95% CI −1.34; 0.06) and android area (−1.08: 95% CI −1.98; −0.19) (Table 4).

Significant differences between groups were noted (ANOVA Group, large effect size) in the level of BM (η^2^ = 0.25, *p* = 0.001), BMI (η^2^ = 0.27, *p* = 0.001), LBM (η^2^ = 0.16, *p* = 0.014), total body fat mass (η^2^ = 0.31, *p* < 0.001), percentage of fat in total body mass (η^2^ = 0.21, *p* = 0.005) and the percentage of fat in the trunk (η^2^ = 0.23, *p* = 0.003) as well as abdominal areas (η^2^ = 0.21, *p* = 0.004), and furthermore, in the percentage of fat in the android/gynoid sections (η^2^ = 0.15, *p* = 0.020) (Table 3). 

Both before the 1st WBC application as well as after 10 and after 20 WBC treatments, the level of these variables was significantly higher (post-hoc, *p* < 0.05) in the MetS group compared to the group of healthy women (Table 4).

No influence of Group×Treatment factor interaction on the level of indices was demonstrated (Table 3 and Table 4).

#### 3.1.2. Circumferences

Significant influence of WBC (ANOVA Treatment, large effect size) on changes in WC (η^2^ = 0.33, *p* < 0.001), AC (η^2^ = 0.22, *p* = 0.001), HC (η^2^ = 0.26, *p* < 0.001) and WHtR (η^2^ = 0.33, *p* < 0.001) was noted (Table 3).

In both groups, there was a significant decrease (*post-hoc*, *p* < 0.05) in WC and WHtR after 10 WBC (−2.17: 95% CI −3.78; −0.56 and −0.01: 95% CI −0.02; −0.00 as well as −1.28: 95% CI −2.30; −0.25 and −0.01: 95% CI −0.01; 0.00, respectively, in the MetS and healthy groups), and 20 WBC treatments (−1.96: 95% CI −3.38; −0.55 and −0.01: 95% CI −0.02; −0.00 as well as −1.57: 95% CI −2.63; −0.50 and −0.01: 95% CI −0.02; 0.00, respectively, in the MetS and healthy groups), and a significant decrease (post-hoc, *p* < 0.05) in AC only after 20 WBC sessions (−2.47: 95% CI −5.25; −0.30 and −2.17: 95% CI −3.69; −0.66, respectively, in the MetS and healthy groups). A significant reduction (post-hoc, *p* < 0.05) in HC was also found in the group of healthy women after 10 WBC (−1.04: 95% CI −1.86; −0.22) and 20 WBC sessions (−1.79: 95% CI −2.54; −1.05) (Table 4).

Significant differences between groups were noted (ANOVA Group, large effect size) in the level of WC (η^2^ = 0.31, *p* = 0.002), AC (η^2^ = 0.27, *p* = 0.004), HC (η^2^ = 0.27, *p* = 0.004) and WHtR (η^2^ = 0.30, *p* = 0.002) (Table 3).

In all measurements, WC, AC, HC and WHtR were significantly higher (post-hoc, *p* < 0.05) in the MetS group, compared to the group comprising healthy women (Table 4).

No influence of Group × Treatment factor interaction on the level of analysed indices was demonstrated (Table 3 and Table 4).

#### 3.1.3. Skinfolds

Significant influence of WBC was noted (ANOVA Treatment, large effect size) on changes in abdominal thickness (η^2^ = 0.17, *p* = 0.002) and triceps skinfolds (η^2^ = 0.22, *p* < 0.001) (Table 3).

The thickness of the triceps skinfold in both groups decreased significantly (post-hoc, *p* < 0.05) after 20 WBC procedures (−0.54: 95% CI −1.09; 0.01 and −1.01: 95% CI −1.82; −0.20, respectively, in the MetS and healthy groups). In the group of healthy women, the thickness of the abdominal skinfold was significantly (post-hoc, *p* < 0.05) lower after 10 WBC (−1.98: 95% CI −4.05; 0.08) and 20 WBC treatments (−2.56: 95% CI −4.75; −0.36) (Table 4).

Significant differences between groups were noted (ANOVA Group) in abdominal thickness (η^2^ = 0.13, *p* < 0.030) and triceps skinfolds (η^2^ = 0.22, *p* = 0.003) (Table 3).

For all measurements, the thickness of abdominal and triceps skinfolds was significantly greater (post-hoc, *p* < 0.05) in the MetS group compared to healthy women (Table 4).

No influence of Group × Treatment factor interaction on the level of analysed indices was demonstrated (Table 3 and Table 4).

### 3.2. Irisin, IL-6 and CRP Concentrations during the Application of Whole-Body Cryotherapy

**Figure 1:**
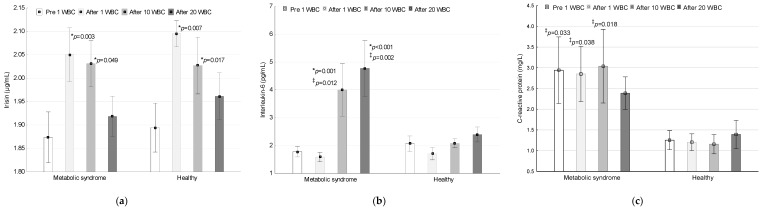
Comparison of concentrations: (**a**) Plasma irisin; (**b**) plasma interleukin-6; (**c**) serum C-reactive protein in the group with metabolic syndrome and in the group of healthy women during the application of whole-body cryotherapy (WBC) procedures. Data presented as mean ± standard deviation; * statistically significant differences compared to values prior to 1st WBC session (*p* < 0.05); ‡ statistically significant differences between groups (*p* < 0.05).

In Figure 1, a comparison of irisin, IL-6 and CRP concentrations in the metabolic syndrome group and in the group of healthy women during whole-body cryotherapy implementation is shown.

#### 3.2.1. Irisin

Irisin concentration in the plasma of women with MetS was 1.87 ± 0.24 µg/mL, 2.05 ± 0.25 µg/mL, 2.03 ± 0.22 µg/mL and 1.92 ± 0.19 µg/mL before and after the 1st WBC session, as well as after the 10th and 20th WBC treatments. In the group of healthy women, the respective values were 1.89 ± 0.22 µg/mL, 2.10 ± 0.12 µg/mL, 2.03 ± 0.26 µg/mL and 1.96 ± 0.21 µg/mL. There were no differences within the groups related to irisin concentration (ANOVA Group; *p* = 0.546, F = 0.37, η^2^ = 0.01, 1-β = 0.09). A significant effect of WBC on irisin concentration was found (ANOVA Treatment; *p* < 0.001, F = 6.76, η^2^ = 0.16, 1-β = 0.97). There was no significant interaction of Group×Treatment factors (ANOVA, *p* = 0.948, F = 0.12, η^2^ < 0.01, 1-β = 0.07).

In the group of women with MetS, the concentration of irisin after 1 WBC (post-hoc, *p* = 0.007) and 10 WBC sessions (post-hoc, *p* = 0.017) was significantly higher than the baseline value. The differences totalled 0.18 (95% CI 0.03; 0.32) and 0.16 (95% CI 0.02; 0.34), respectively. Similarly, in the group of healthy women, after 1 WBC (post-hoc, *p* = 0.003) and 10 WBC sessions (post-hoc, *p* = 0.049), a higher concentration of irisin was detected compared to baseline (respectively, by 0.20: 95% CI 0.09; 0.31 and 0.13: 95% CI 0.01; 0.28). The concentration of irisin after 20 WBC treatments was comparable to baseline (post-hoc, *p* > 0.05), the differences totalling 0.04 (95% CI −0.8; 0.17) and 0.07 (95% CI −0.03; 0.16) in the MetS and health-women groups, respectively.

#### 3.2.2. Interleukin-6

The concentration of IL-6 in the plasma of women with MetSwas 1.78 ± 0.84 pg/mL, 1.58 ± 0.72 pg/mL, 4.00 ± 4.11 pg/mL and 4.77 ± 4.35 pg/mL before WBC 1, after WBC 1, after WBC 10 and after WBC 20, respectively. In the group of healthy women, the respective values were 2.06 ± 1.17 pg/mL, 1.70 ± 0.94 pg/mL, 2.08 ± 0.68 pg/mL and 2.40 ± 1.14 pg/mL. There was a significant group effect (ANOVA Group; *p* = 0.045, F = 4.33, η^2^ = 0.11, 1-β = 0.52), WBC (ANOVA Treatment; *p* < 0.001, F = 7.20, η^2^ = 0.17, 1-β = 0.98) and the interaction of the Group×Treatment factors (ANOVA, *p* = 0.009, F = 4.03, η^2^ = 0.10, 1-β = 0.83) on the concentration of IL-6 in the plasma.

The IL-6 concentration increased significantly after the 10 WBC (post-hoc, *p* = 0.001) and after the 20 WBC procedures (post-hoc, *p* < 0.001), but only in women with MetS (2.22: 95% CI 0.15; 4.29 and 2.99: 95% CI 0.73; 5.26, respectively). The change in IL-6 concentration after 1 WBC application in the MetS group was not statistically significant (post-hoc, *p* > 0.05) and totalled −0.19 (95% CI −0.38; −0.01). In the group of health women, changes in IL-6 concentrations were non-significant (post-hoc, *p* > 0.05) and totalled −0.36 (95% CI −0.68; −0.05), 0.01 (95% CI −0.66; 0.69) and 0.33 (95% CI −0.32; 0.99), after 1, 10 and 20 WBC treatments, respectively.

Baseline plasma levels of IL-6 were similar in both groups (*p* > 0.05), while after WBC 10 (*p* = 0.012) and WBC 20 (*p* = 0.002), they were higher in women with MetS compared to the healthy subjects.

#### 3.2.3. C-Reactive Protein

The CRP concentration in the serum of women with MetS was 2.94 ± 3.50 mg/L, 2.85 ± 2.90 mg/L, 3.04 ± 3.86 mg/L and 2.39 ± 1.72 mg/L (respectively) before and after WBC 1, after WBC 10 and after WBC 20. In the group of healthy women, the respective values were 1.25 ± 0.97 mg/L, 1.20 ± 0.85 mg/L, 1.16 ± 0.99 mg/L and 1.39 ± 1.44 mg/L. There were significant group-related differences in CRP concentration (ANOVA Group; *p* = 0.030, F = 5.13, η^2^ = 0.13, 1-β = 0.60), however, no effects of WBC (ANOVA Treatment; *p* = 0.880, F = 0.22, η^2^ = 0.01, 1-β = 0.09) or Group×Treatment (ANOVA *p* = 0.460, F = 0.87, η^2^ = 0.02, 1-β = 0.23) were noted on the CRP concentration in these groups.

The CRP concentration in the serum before (post-hoc, *p* = 0.033), after the 1 WBC (post-hoc, *p* = 0.038) and after the 10 WBC session (post-hoc, *p* = 0.018) was significantly higher in the MetS group compared to the healthy women. After 20 WBC treatments, the CRP concentration was comparable in both groups (*p* > 0.05).

### 3.3. Correlations

In the MetS group and in the group of healthy women, the correlation between the concentration of irisin, IL-6, and between changes in their concentration and statistically significant changes in somatic constitution indices was assessed.

In the MetS group, a significant negative correlation was found between the change in irisin concentration after 10 WBC treatments, and between the change in HC (r = −0.84, *p* < 0.05). Furthermore, between the change in irisin concentration after 20 WBC sessions and the change in body mass (r = −0.66, *p* < 0.05), as well as between the concentration of irisin after 20 WBC applications and the change in total fat percentage (r = −0.48, *p* < 0.05). There was also a significant negative correlation between the change in IL-6 concentration after 10 WBC procedures and a change in WHR (r = −0.65, *p* < 0.05), WHtR (r = −0.64, *p* < 0.05) and WC (r = −0.63, *p* < 0.05). No such correlations were found in the group of healthy women.

## 4. Discussion

A factor strongly affecting the human body is cryogenic temperature used during WBC [30]. Our study was the first to show that WBC can effectively support the treatment of abdominal obesity in menopausal women. It has been proven that the first beneficial changes occur after 10 WBC treatments and apply to both menopausal women with MetS and without severe metabolic disorders. Increasing the number of WBC treatments to 20 increased the beneficial changes in body composition among menopausal women.

In our study, 20 WBC treatments were used, which were applied daily in 4 series of 5 treatments, between which there were 2-day intervals (weekends). The procedures were performed among menopausal women in whom MetS occurs more often than in young women and men [6,16]. On the other hand, previous research on the influence of WBC on the body of obese individuals, conducted by other scientists, concerned men [24,25,26,28]. In order to eliminate additional factors that may affect the obtained results [51], during the period of using WBC, the women in our study did not change their diets or physical activity, which they declared to be at a low or sufficient level [46].

The basic method of assessing overweightness and obesity is BMI [1]. In our study, in both groups, after 20 WBC treatments, the body mass of the participants decreased and, consequently, the BMI value also experienced a significant reduction. After the procedures, the BMI level in 26 subjects was lower than before cryotherapy. But only in one of the participants undergoing therapy did BMI normalisation occur. However, the knowledge of BMI values does not allow determination of adipose tissue distribution.

Although according to current criteria, abdominal obesity is not a necessary condition for the diagnosis of MetS [10,11,12,13], besides arterial hypertension, it is the most common disorder in this disease [19]. Abdominal obesity leads to life-threatening consequences. It influences the development of insulin resistance and disorders of lipid metabolism, leading to excessive oxidation of fatty acids and consequently, to oxidative stress and the formation of atherosclerotic plaques, the development of hypertension and chronic inflammation [9,18,19,52].

Visceral fat content can be estimated by measuring waist circumference and determining the waist-to-hip ratio as well as the waist-to-body height ratio [53,54]. In our study, a waist circumference above 88 cm, which was one of the MetS diagnostic criteria according to NCEP - ATP III, was present in as many as 45.9% of all participants. Among women with MetS, as many as 73.7% met this criterion. It was found that after 10 WBC treatments there was a reduction in WC and WHtR in both of the compared groups, and in healthy menopausal women the hip circumference and abdominal skinfold thickness were also reduced. After 20 WBC procedures, in both groups, these favourable changes intensified. In addition, after 20 WBC units, in both groups, the abdominal circumference and the thickness of the skin fold above the triceps muscle decreased compared to the value measured before cryotherapy. Based on the values of confidence intervals, it may be concluded that in these cases, the direction of changes was uniform in each group. A large effect size regarding the changes was also found for these variables, the greatest in WC and WHtR. The results obtained prove the beneficial influence of WBC in the battle against obesity. Also, in young obese men, the beneficial effect of 20 WBC treatments can been seen in the reduction of WC and HC [28]. However, in these studies, the influence of diet and physical activity on the results cannot be ignored. Researchers advised participants not to change their habits, however, this was not controlled during the application of WBC [28]. In our study, diet and physical activity were monitored during the use of WBC, and it was found that they were not significantly modified in any of the groups. This excludes the possibility of these factors affecting our obtained results.

In our study we assessed the influence of WBC on body composition and adipose tissue distribution using the dual DXA. It was found that in both groups including menopausal women, weight loss after the 20th WBC session was the result of a significant reduction in body fat mass. Analysing the changes in the distribution of adipose tissue in individual body segments, it was noted that in both groups, the percentage of adipose tissue in the lower limbs decreased. In the group of women with MetS, the beneficial effects of cryotherapy also consisted in a significant reduction in the percentage of body fat in the entire trunk area, but also in the percentage of abdominal fat. The analysis of results indicates a one-way, clinically beneficial (large or medium effect size) changes concerning the majority of the analysed variables.

The number of exposures to cryogenic temperatures seems to be significant in influencing changes regarding body composition. As in the case of women in our study, the use of 10 WBCs in young men did not affect the total body mass or the percentage of body fat [55]. In this study [55], WBC treatments lasting 2 min at −110 °C were performed twice a day for 5 consecutive days. Changes in body composition after the application of 10 WBC treatments performed daily for 3 min at a temperature of −110°C in obese men around the age of 40, were also not found by Ziemann et al. [25], who compared the results of people with lower (VO_2max_ 25.7 ± 2.0 mL/kg/min, BMI 34.0 ± 2.5 kg/m^2^) and higher cardiorespiratory fitness (VO_2max_ 43.3 ± 3.0 mL/kg/min, BMI 31.4. ± 2.0 kg/m^2^). Also, Lubkowska et al. [24] did not find any significant changes in body composition as a result of WBC application, although the treatments were supported by physical training. In these studies, the volunteers were obese men (40 ± 4 years, BMI 30.39 ± 4.31 kg/m^2^) who participated in a 6-month training cycle with moderate intensity exercises 3 times a week. In the 2nd and 6th month of training, they were additionally subjected to 20 WBC treatments (3 min, −130 °C). Waist and hip circumferences and WHR did not change significantly. There were also no changes to body fat mass, subcutaneous fat mass, visceral fat mass, LBM, skeletal muscle mass, or the percentage of these components during the study [24]. However, in research involving younger men with low physical activity, both obese (29.08 ± 4.19 y, BMI 36.23 ± 8.13 kg/m^2^) and with a normative body mass (22.00 ± 2.45 y, BMI 23.58 ± 2.00 kg/m^2^), as a result of 20 WBC exposures (2–3 min, −120 °C), significant decreases were noted in BM, fat mass and percentage. In the group of obese men, this resulted in a decrease in BMI [28].

The results of our research indicate the need for at least 20 WBC treatments in a series to achieve beneficial changes in body composition among menopausal women. In our study, we found that in both groups of women, WBC treatments had a similar effect, which is indicated by the lack of statistical significance in the assessment of the interaction of the Group x Treatment factors, with significant impact of the Treatment factor on the level of the analysed variables. Such results, i.e., the beneficial effects of WBC on body composition in both groups, are not surprising when considering the results of previous studies with the participation of men, in which positive changes in body composition of the participants were also obtained in two groups significantly different in the level of fat and BMI before beginning the procedures [28]. However, we expected greater clinical effects, especially in the group of women with MetS. Increasing the number of WBC treatments in a series is likely to intensify the clinical effect. However, this requires further investigation.

In our study, beneficial changes in body composition in the group of women with MetS were associated with increases in blood irisin levels. We found that in both groups, the level of irisin increased significantly after WBC 1 and WBC 10 (large effect size).

The primary physiological mechanism, preventing heat loss in response to cryogenic temperatures, is the stimulation of the sympathetic nervous system and vasoconstriction in the dermis and subcutaneous tissue, as well as may induce shivering thermogenesis. During the second phase, following the procedure, a vasodilation and an increase in perfusion take place [29]. Muscle cell contractions induce the expression of the *FNDC5* gene by increasing PGC1-α levels, which affects the synthesis of the FNDC5 protein and the release of irisin into the bloodstream. Irisin is a thermogenic adipomyokine [39]. It has been shown that irisin increases the level of the UCP-1 uncoupling protein, a slight increase in its concentration causes browning of white adipose tissue, which affects the thermogenesis process [36]. UCP-1 mediates proton transport across the inner mitochondrial membrane bypassing ATP synthase. Therefore, energy is dissipated in the form of heat [56]. The release of heat energy leads to the rapid depletion of cellular storage substrates, which is associated with weight loss. It has been shown that exogenous irisin stimulates the activation of lipoprotein lipase and triglyceride lipolysis, enhances lipid metabolism and glucose uptake by cells, and lowers lipid synthesis in mice [57,58].

Cross-correlations showed that greater increases in irisin levels in our study resulted in greater weight loss, and higher levels of this hormone were associated with greater reductions in body fat percentage among women with MetS as a result of cryotherapy. Thus, it has been shown that favourable changes in body composition may be associated with the induction of changes in irisin concentration by cryogenic temperatures. However, it is surprising that there were no changes in the concentration of irisin after 20 WBC treatments, which has already been reported by us [27]. A possible reason for such a reaction is the adaptation to cold and the weakening of shivering thermogenesis as a probable factor inducing an increase in irisin concentration, which was reported as subjective sensations by participants.

Dulian et al. [26], who used WBC procedures (3 min, −110 °C) in obese men (BMI > 30 kg/m^2^) aged 38 ± 9 years, found that the change in irisin concentration in response to WBC depends on the level of physical fitness. Based on the maximum oxygen uptake, the subjects were divided into HFL—high fitness level (VO_2max_ > 35 mL/kg/min) and LFL—low fitness level (VO_2max_ < 35 mL/kg/min) groups. After 10 WBC sessions, a slight decrease in irisin concentration was found in the HFL group. However, in the LFL group, 10 WBC treatments have been found to increase the concentration of irisin in the blood by about 18% [26]. In the research by Sliwicka et al. [43], a significant increase in the concentration of irisin in the blood was found in young men with LFL (VO_2max_ < 43 mL/kg/min) in response to the 10th WBC procedure. In young men, the irisin concentration did not change significantly after the 1st WBC treatment or after the 10th WBC series [43]. In our study, women from both groups were characterised by low or moderate physical activity, none of the women practiced high-level physical activity.

In the research by Dulian et al. [26], a positive correlation was indicated between the concentration of irisin and the mass and percentage of fat as well as with the visceral fat area, and a negative correlation with the mass of the skeletal muscles. This gave rise to the claim that the main source of irisin in response to cryogenic exposure is subcutaneous adipose tissue [26], although the results of our research do not support this conclusion. In this study, no correlation was demonstrated between irisin levels and adipose tissue or LBM.

The reaction to obesity-related inflammation is the activation of the NF-ĸB transcription factor, which enhances, among others, the production of pro-inflammatory interleukins such as IL-1, IL-6 and TNF-α, and the concentration of C-reactive protein also increases [18,19]. In our study, women with MetS had significantly higher CRP levels compared to healthy menopausal women, with comparable IL-6 and irisin levels. In our study, the group of healthy women comprised participants who did not meet the MetS criteria according to the NCEP-ATP III concept. However, this did not exclude the occurrence of metabolic disorders (less than 3 criteria), which is the probable cause of similar IL-6 and irisin concentration levels in both groups.

WBC treatments did not significantly change the CRP concentration in any of the groups with menopausal women. In contrast, the concentration of IL-6 increased significantly in the MetS group after 10 and 20 WBC applications (large effect size). The anti-inflammatory effect of whole-body cryotherapy is demonstrated by an increase in the concentration of irisin and IL-6 without changes in the concentration of CRP in the blood. As in this study, there were no significant changes in CRP levels after 10 WBC treatments in 20-year-old non-obese men, regardless of their physical fitness level [43]. In contrast, in obese 40-year-old men, both in the LFL and HFL groups, after 10 WBC, the CRP concentration was significantly lower than before the initiation of cryotherapy, which indicates an anti-inflammatory effect of the treatments, and the concentration of IL-6, similar to that in young men [43], did not change [26]. Many of the previously researches indicate the anti-inflammatory effect of WBC [30]. Lubkowska et al. [59] found that in young men, the concentration of IL-6 in the blood is higher than the baseline after both 1 WBC and 10 WBC exposures. However, in this research, the levels of other markers of inflammation were not measured [59].

IL-6 has a pleiotropic effect and may play a pro-and anti-inflammatory role [60]. Diseases and stress-related muscle damage cause a significant increase in the concentration of IL-6 in the blood, with a simultaneous increase in the concentration of other pro-inflammatory cytokines, such as TNF-α or IL-1β. In contrast, exercise that does not cause micro-damage increases the concentration of IL-6, but does not increase the concentration of other pro-inflammatory cytokines [35]. In such a situation, IL-6 shows a metabolic effect stimulating lipolysis and fat oxidation [34]. The persistently elevated concentration of IL-6 in the blood indicates low-grade chronic inflammation, such as, for example, in obese people [18,19]. It has been shown that visceral obesity is associated with high CRP and high IL-6 levels [41]. But low levels of irisin, an anti-inflammatory effect, were found only in people with high CRP, not high levels of IL-6 [41]. In our study we found a significant increase of the levels of IL-6 in the blood of menopausal women with MetS in response to 10 and 20 WBC treatments, but without a concomitant change in CRP levels, indicating the anti-inflammatory effect of IL-6 in this situation. Unfortunately, we did not simultaneously study changes in the concentration of other inflammation markers, such as other pro- and anti-inflamed interleukins, which limits the possibility of unequivocal inference. Nonetheless, similar results were obtained by other researchers who, in young healthy men, found higher than baseline levels of IL-6 in the blood after 5 WBC, 10 WBC and 20 WBC sessions [33]. These changes were accompanied by a simultaneous increase in the concentration of anti-inflammatory IL-10 and a decrease in the concentration of pro-inflammatory IL-1α, without significant changes in the levels of TNF-α, IL-1β and IL-12 [33]. In professional tennis players, it was found that 10 WBC exposures used twice a day for 5 days in conjunction with moderate-intensity training, the concentration of IL-6 in the blood increases, but at the same time, the concentration of TNF-α decreases [61]. In turn, in obese men, the reaction to WBC was influenced by their fitness level. In people with low fitness level, after 10 WBC sessions, there was a decrease in the concentration of IL-6 and TNF-α, without affecting the change in people with high levels of fitness [25]. The results of our study showed a beneficial relationship between the increase in IL-6 concentration and changes in body composition in menopausal women with MetS. Showing statistically significant correlations, it was found that a greater increase in IL-6 concentration was associated with a greater decrease in waist circumference and a greater decrease in WHR and WHtR.

The results of our study indicate the stimulating effect of cryogenic temperatures on the secretion of irisin and IL-6 and indirectly on their participation in the modulation of metabolic changes leading to beneficial changes in body composition of menopausal women. However, the research has its limitations. They mostly regard the assessment of changes in blood cytokine levels. This limits the possibility of indicating the source of their secretion. Subsequent research should be focused on the evaluation of mRNA expression for pro- and anti-inflammatory interleukins, as well as adipocytokines and UCP-1 both in blood mononuclear cells, as well as in myocytes and adipocytes in response to WBC treatments applied in subjects with varying degrees of fat and including both sexes. In our study, we obtained significant changes as an effect of 20 WBC procedures, indicating the clinically beneficial influence of these treatments on body composition (reduction of abdominal obesity) in menopausal women. However, it seems that increasing the number of treatments would increase the clinical effects. Considering the significant influence of changes in physical activity and diet on body composition, the idea of our study was to exclude the influence of these factors and to check to what extent cryotherapy treatments alone are able to induce beneficial changes in body composition among menopausal women. The volunteers did not change their previous behaviour, nor did they participate in clinical therapy, they were not subjected to pharmacotherapy, and did not change their physical activity or diet during the trial. More research is needed including a greater number of volunteers that would undergo more WBC treatments. Subsequent research should include people representing different levels of physical activity and the researchers should simultaneously assess the effects of WBC and diet modification.

## 5. Conclusions

Whole-body cryotherapy, assuming the application of 20 treatments in the series, reduces abdominal obesity in menopausal women indirectly through the secretion of irisin and IL-6, and can be used as adjunctive therapy in the treatment of metabolic syndrome. Our conclusion is limited to menopausal women with low-moderate physical activity for whom its level as well as diet were not changed during the treatment.

## Figures and Tables

**Table 1:** Age and medical qualification.

Variable	Metabolic Syndrome	Healthy	Total	Min-Max
Age (years)	61.53 ± 3.99	60.28 ± 3.63	60.92 ± 3.82	55.00–67.00
Erythrocytes (10^6^/µL)	4.62 ± 0.22	4.59 ± 0.24	4.61 ± 0.23	4.20–5.10
Haemoglobin (g/dL)	13.89 ± 0.55	13.94 ± 0.55	13.92 ± 0.55	13.20–15.30
Haematocrit (%)	40.82 ± 1.75	41.14 ± 1.62	40.98 ± 1.67	38.60–44.60
ESR (mm/h)	19.58 ± 10.67	16.11 ± 9.65	17.89 ± 10.19	2.00–43.00
Platelets (10^3^/µL)	246.53 ± 77.69	254.89 ± 56.59	250.59 ± 67.44	61.00–457.00
Leukocytes (10^3^/µL)	6.16 ± 1.25 *	5.29 ± 1.06	5.74 ± 1.23	3.82–8.53
Neutrophils (10^3^/µL)	3.04 ± 0.86	2.60 ± 0.57	2.83 ± 0.76	1.60–5.40
Lymphocytes (10^3^/µL)	2.24 ± 0.56	1.92 ± 0.50	2.08 ± 0.55	1.10–3.40
Monocytes (10^3^/µL)	0.48 ± 0.11	0.50 ± 0.14	0.49 ± 0.12	0.26–0.84
Eosinophils (10^3^/µL)	0.18 ± 0.09	0.17 ± 0.09	0.18 ± 0.09	0.06–0.42
Basophils (10^3^/µL)	0.02 ± 0.03	0.04 ± 0.04	0.03 ± 0.03	0.00–0.10
HbA_1C_ (%)	5.84 ± 0.28 *	5.67 ± 0.29	5.75 ± 0.29	5.20–6.50
Insulin (µIU/mL)	12.12 ± 5.65 *	8.27 ± 2.50	10.25 ± 4.77	4.40–28.20
HOMA-IR	3.03 ± 1.52 *	1.92 ± 0.61	2.49 ± 1.29	0.95–7.19
TCHOL (mg/dL)	213.50 ± 37.57	223.56 ± 35.55	218.39 ± 6.45	157.90–295.28
LDL (mg/dL)	127.78 ± 37.13	137.81 ± 35.04	132.66 ± 35.99	57.13–214.12
AIP (log_10_TG/HDL)	0.38 ± 0.23 *	0.22 ± 0.20	0.30 ± 0.23	-0.15–0.93
Homocysteine (µmol/L)	13.15 ± 1.97	12.26 ± 1.71	12.72 ± 1.88	9.00–17.00
hsCRP (mg/L)	2.94 ± 3.50 *	1.25 ± 097	2.12 ± 2.70	0.27–15.89

Values are means ± SD; ESR: erythrocyte sedimentation rate, HbA_1c_: glycated haemoglobin, HOMA-IR: homeostasis model assessment of insulin resistance, TCHOL: total cholesterol, LDL: low density lipoproteins, AIP: atherogenic index of plasma, CRP: C-reactive protein; * *p* < 0.05, significant differences: metabolic syndrome group vs. healthy (*t*-test or Mann–Whitney U test).

**Table 2:** Diagnostics of metabolic syndrome in accordance with NCEP-ATP III in compared groups.

Group	WC (cm)	TG (mg/dL)	HDL (mg/dL)	Glucose (mg/dL)	SBP (mmHg)	DBP (mmHg)
Metabolic syndrome	96.24 ± 9.91 *	130.65 ± 41.40	55.50 ± 12.17 *	102.39 ± 9.81 *	127.63 ± 17.27	82.63 ± 7.88
Healthy	84.22 ± 8.71	111.47 ± 38.47	65.06 ± 14.11	92.93 ± 6.94	120.17 ± 16.78	77.83 ± 7.69
Total	88.81 ± 8.58	121.32 ± 40.63	60.15 ± 13.84	97.79 ± 9.69	124.00 ± 17.22	80.30 ± 8.06
Min-Max	73.60−109.80	63.88−215.25	41.41−91.72	73.60−109.80	90−150	60−90
**Number of people fulfilling given criterion for diagnosis of metabolic syndrome NCEP-ATP III**						
Metabolic syndrome	14 (73.7%)	6 (31.6%)	7 (36.8%)	15 (78.9%)	10 (52.6%)	7 (36.8%)
Healthy	3 (16.7%)	3 (16.7%)	1 (5.6%)	3 (16.7%)	6 (33.3%)	4 (22.2%)
Total	17 (45.9%)	9 (24.3%)	8 (21.6%)	18 (48.6%)	16 (43.2%)	11 (29.7%)
**Number of metabolic syndrome criteria NCEP-ATP III fulfilled by volunteers**						
	**Healthy**			**Metabolic syndrome**		
Number of criteria	None	One	Two	Three	Four	Five
Number of people	4 (10.8%)	10 (27.0%)	4 (10.8%)	11 (29.7%)	4 (10.8%)	4 (10.8%)

Values are means ± SD; NCEP-ATP III: National Cholesterol Education Program-Adult Treatment Panel III, WC: waist circumference, TG: triglycerides, HDL: high density lipoproteins, SBP: systolic blood pressure, DBP: diastolic blood pressure; * *p* < 0.05, significant differences: metabolic syndrome group vs. healthy (*t*-test or Mann–Whitney U test).

**Table 3:** Assessment regarding the effects of whole-body cryotherapy (WBC) treatments on changes in body composition markers-analysis of variance with repeated measures (ANOVA).

Variable	Group (G)				Treatment (T)				Interaction G×T			
	*p*	F	Power Test 1-β	Effect Size η^2^	*p*	F	Power Test 1-β	Effect Size η^2^	*p*	F	Power Test 1-β	Effect Size η^2^
**Body composition**												
Body Mass (kg)	0.001	11.98	0.92	0.25	0.001	7.31	0.93	0.17	0.780	0.25	0.09	0.01
BMI (kg/m^2^)	0.001	12.62	0.93	0.27	0.001	7.47	0.93	0.18	0.824	0.19	0.08	0.01
Lean Body Mass (kg)	0.014	6.74	0.71	0.16	0.760	0.28	0.09	0.01	0.623	0.48	0.12	0.01
Total Body Fat (kg)	<0.001	15.49	0.97	0.31	0.001	8.42	0.96	0.19	0.954	0.05	0.06	<0.01
Total Body Fat (%)	0.005	9.04	0.83	0.21	0.036	3.50	0.63	0.09	0.840	0.18	0.08	<0.01
Arm Fat (%)	0.216	1.59	0.23	0.04	0.221	1.54	0.32	0.04	0.976	0.02	0.05	<0.01
Leg Fat (%)	0.091	3.01	0.39	0.08	0.006	5.59	0.84	0.14	0.986	0.01	0.05	<0.01
Trunk Fat (%)	0.003	10.32	0.88	0.23	0.113	2.25	0.44	0.06	0.654	0.43	0.12	0.01
Android Fat (%)	0.004	9.52	0.85	0.21	0.049	3.06	0.57	0.08	0.484	0.73	0.17	0.02
Gynoid Fat (%)	0.098	2.88	0.38	0.08	0.277	1.31	0.27	0.04	0.353	1.06	0.23	0.03
A/G	0.020	5.94	0.66	0.15	0.314	1.18	0.25	0.03	0.792	0.23	0.09	0.01
**Circumferences**												
WC (cm)	0.002	11.94	0.91	0.31	<0.001	13.54	0.99	0.33	0.516	0.67	0.16	0.02
AC (cm)	0.004	9.95	0.86	0.27	0.001	7.52	0.93	0.22	0.471	0.76	0.17	0.03
HC (cm)	0.004	9.93	0.86	0.27	<0.001	9.67	0.98	0.26	0.372	1.01	0.22	0.04
WHR	0.124	2.52	0.35	0.09	0.080	2.71	0.51	0.09	0.363	1.03	0.22	0.04
WHtR	0.002	11.60	0.91	0.30	<0.001	13.19	0.99	0.33	0.521	0.66	0.16	0.02
**Skinfold thickness**												
Abdominal (mm)	0.030	5.12	0.59	0,13	0.002	6.94	0.91	0.17	0.052	3.09	0.58	0.08
Subscapular (mm)	0.214	1.60	0.23	0.04	0.643	0.45	0.12	0.01	0.572	0.56	0.14	0.02
Triceps (mm)	0.003	9.96	0.87	0.22	<0.001	9.84	0.98	0.22	0.357	1.04	0.23	0.03

*p* < 0.05: statistically significant differences; BMI: body mass index, A/G: android-to-gynoid percentage fat ratio; WC: waist circumference, AC: abdominal circumference, HC: hip circumference, WHR: waist-hip ratio; WHtR: waist–height ratio.

**Table 4:** Changes in body composition, circumferences and fatfold thickness as an effect of applying whole-body cryotherapy (WBC) treatments.

	Metabolic	Syndrome				Healthy				
Variable	Pre 1 WBC	After 10 WBC	After 20 WBC	Δ 10 WBC	Δ 20 WBC	Pre 1 WBC	After 10 WBC	After 20 WBC	Δ 10 WBC	Δ 20 WBC
	Mean ± SD	Mean ± SD	Mean ± SD	Mean (95% CI)	Mean (95% CI)	Mean ± SD	Mean ± SD	Mean ± SD	Mean (95% CI)	Mean (95% CI)
**Body composition**										
Body Mass (kg)	77.36 ± 11.95	76.97 ± 12.06	76.80 ± 12.13 *	−0.38 (−0.89; 0.12)	−0.56 (−1.20; 0.08)	66.32 ± 6.23 ^‡^	66.06 ± 6.50 ^‡^	65.66 ± 6.32 *^,^^‡^	−0.27 (−0.64; 0.11)	−0.67 (−0.98; −0.35)
BMI (kg/m^2^)	30.09 ± 4.98	29.93 ± 4.93	29.86 ± 4.94 *	−0.16 (−0.36; 0.03)	−0.24 (−0.49; 0.02)	25.50 ± 2.37 ^‡^	25.39 ± 2.45 ^‡^	25.24 ± 2.43 *^,^^‡^	−0.11 (−0.25; 0.04)	−0.25 (−0.37; 0.14)
Lean Body Mass (kg)	44.19 ± 4.75	44.14 ± 5.11	44.21 ± 4.83	−0.47 (−0.59; 0.50)	0.24 (−0.43; 0.48)	40.76 ± 3.31 ^‡^	40.68 ± 3.28 ^‡^	40.52 ± 2.96 ^‡^	−0.08 (−0.43; 0.27)	−0.24 (−0.65; 0.17)
Total Body Fat (kg)	34.42 ± 8.58	34.15 ± 8.55	33.92 ± 8.81 *	−0.27 (−0.61; 0.08)	−0.50 (−0.93; −0.07)	25.52 ± 4.08 ^‡^	25.32 ± 4.33 ^‡^	25.01 ± 4.33 *^,^^‡^	−0.20 (−0.53; 0.12)	−0.51 (−0.89; −0.12)
Total Body Fat (%)	42.42 ± 4.32	42.21 ± 4.45	41.93 ± 4.59 *	−0.21 (−0.69; 0.28)	−0.48 (−0.91; −0.05)	38.38 ± 3.48 ^‡^	38.22 ± 3.63 ^‡^	38.07 ± 3.54 ^‡^	−0.16 (−0.56; 0.25)	−0.31 (−0.84; 0.22)
Arm Fat (%)	42.67 ± 4.37	43.18 ± 3.96	42.87 ± 4.18	0.51 (−0.42; 1.44)	0.20 (−0.52; 0.92)	41.06 ± 3.88	41.51 ± 4.44	41.14 ± 4.02	0.44 (−0.55; 1.44)	0.08 (−0.73; 0.89)
Leg Fat (%)	39.62 ± 4.99	39.40 ± 5.08	39.07 ± 5.01 *	−0.22 (0.73; 0.29)	−0.55 (−1.01; −0.09)	36.99 ± 3.97	36.82 ± 4.13	36.49 ± 4.07 *	−0.17 (−0.52; 0.18)	−0.51 (−1.10; 0.09)
Trunk Fat (%)	46.54 ± 5.08	46.21 ± 5.42	45.90 ± 5.69 *	−0.33 (−1.01; 0.34)	−0.64 (−1.34; 0.06)	41.02 ± 4.88 ^‡^	40.72 ± 4.85 ^‡^	40.75 ± 4.89 ^‡^	−0.31 (−0.91; 0.30)	−0.27 (−0.98; 0.43)
Android Fat (%)	48.98 ± 5.93	48.40 ± 6.34	47.90 ± 6.57 *	−0.58 (−1.39; 0.22)	−1.08 (−1.98; −0.19)	42.74 ± 5.58 ^‡^	42.25 ± 5.82 ^‡^	42.34 ± 5.61 ^‡^	−0.49 (−1.36; 0.38)	−0.39 (−1.45; 0.66)
Gynoid Fat (%)	44.24 ± 4.66	43.90 ± 4.67	43.69 ± 4.90	−0.34 (−0.91; 0.23)	−0.55 (−1.14; 0.05)	41.0 ± 3.40	41.69 ± 3.58	41.55 ± 3.74	0.09 (−0.47; 0.66)	−0.05 (−0.68; 0.58)
A/G	1.11 ± 0.10	1.10 ± 0.09	1.10 ± 0.11	−0.01 (−0.03; 0.01)	−0.01 (−0.04; 0.02)	1.03 ± 0.11^‡^	1.01 ± 0.12 ^‡^	1.02 ± 0.11 ^‡^	−0.01 (−0.03; 0.00)	−0.01 (−0.03; 0.01)
**Circumferences**										
WC (cm)	96.24 ± 9.91	94.06 ± 8.25 *	94.27 ± 9.96 *	−2.17 (−3.78; −0.56)	−1.96 (−3.38; −0.55)	84.22 ± 8.71 ^‡^	82.94 ± 8.46 *^,^^‡^	82.66 ± 8.27 *^,^^‡^	−1.28 (−2.30; −0.25)	−1.57 (−2.63; −0.50)
AC (cm)	104.94 ± 10.98	103.16 ± 9.76	102.46 ± 10.76 *	−1.77 (−4.82; 1.27)	−2.47 (−5.25; −0.30)	93.91 ± 7.40 ^‡^	93.54 ± 7.6 ^‡^	91.73 ± 7.77 *^,^^‡^	−0.37 (−1.66; 0.93)	−2.17 (−3.69; −0.66)
HC (cm)	112.06 ± 11.71	111.21 ± 11.90	111.13 ± 12.24	−0.85 (−2.07; 0.36)	−0.94 (−2.30; 0.42)	102.13 ± 6.18 ^‡^	101.09 ± 5.43 *^,^^‡^	100.34 ± 5.4 *^,^^‡^	−1.04 (−1.86; −0.22)	−1.79 (−2.54; −1.05)
WHR	0.86 ± 0.04	0.85 ± 0.03	0.85 ± 0.04	−0.01 (−0.02; 0.00)	−0.01 (−0.02; −0.00)	0.82 ± 0.06	0.82 ± 0.06	0.82 ± 0.06	0.00 (−0.01; 0.00)	0.00 (−0.01; 0.00)
WHtR	0.59 ± 0.06	0.58 ± 0.05*	0.58 ± 0.06*	−0.01 (−0.02; −0.00)	−0.01 (−0.02; −0.00)	0.52 ± 0.05 ^‡^	0.51 ± 0.05 *^,‡^	0.51 ± 0.05 *^,^^‡^	−0.01 (−0.01; 0.00)	−0.01 (−0.02; 0.00)
**Skinfold thickness**										
Abdominal (mm)	29.44 ± 8.65	29.66 ± 8.26	28.56 ± 8.17	0.22 (−0.63; 1.06)	−0.88 (−1.85; 0.08)	25.17 ± 7.12 ^‡^	23.18 ± 7.08 *^,^^‡^	22.61 ± 6.23 *^,^^‡^	−1.98 (−4.05; 0.08)	−2.56 (−4.75; −0.36)
Subscapular (mm)	24.91 ± 7.95	24.91 ± 7.48	24.96 ± 7.48	0.01 (−0.43; 0.44)	0.06 (−0.66; 0.78)	21.87 ± 6.76	22.24 ± 6.20	21.88 ± 6.38	0.37 (−0.36; 1.11)	0.02 (−0.79; 0.83)
Triceps (mm)	24.31 ± 4.95	24.30 ± 4.73	23.77 ± 4.63 *	−0.01 (−0.45; 0.44)	−0.54 (−1.09; 0.01)	19.84 ± 4.90^‡^	19.41 ± 4.47 ^‡^	18.83 ± 4.05 *^,^^‡^	−0.43 (−1.03; 0.17)	−1.01 (−1.82; −0.20)

SD: standard deviation, CI: confidence interval, **Δ** 10 WBC: difference after 10 WBC compared to pre 1 WBC, **Δ** 20 WBC: difference after 20 WBC compared to pre 1 WBC; BMI: body mass index, A/G: android-to-gynoid percentage fat ratio; WC: waist circumference, AC: abdominal circumference, HC: hip circumference, WHR: waist-hip ratio; WHtR: waist-height ratio; * statistically significant differences compared to values pre 1 WBC (*p* < 0.05); ^‡^ statistically significant differences between groups (*p* < 0.05).
